# Supplementary material for: A synthetic transcription platform for programmable gene expression in mammalian cells
Source: Nat Commun. 2022 Oct 18;13:6167. doi: 10.1038/s41467-022-33287-9 (PMC9579178; doi:10.1038/s41467-022-33287-9)
Supplement: Supplementary file 1 — Supplementary Information [file 41467_2022_33287_MOESM1_ESM.pdf]

Supplementary Figures and Figure Legends

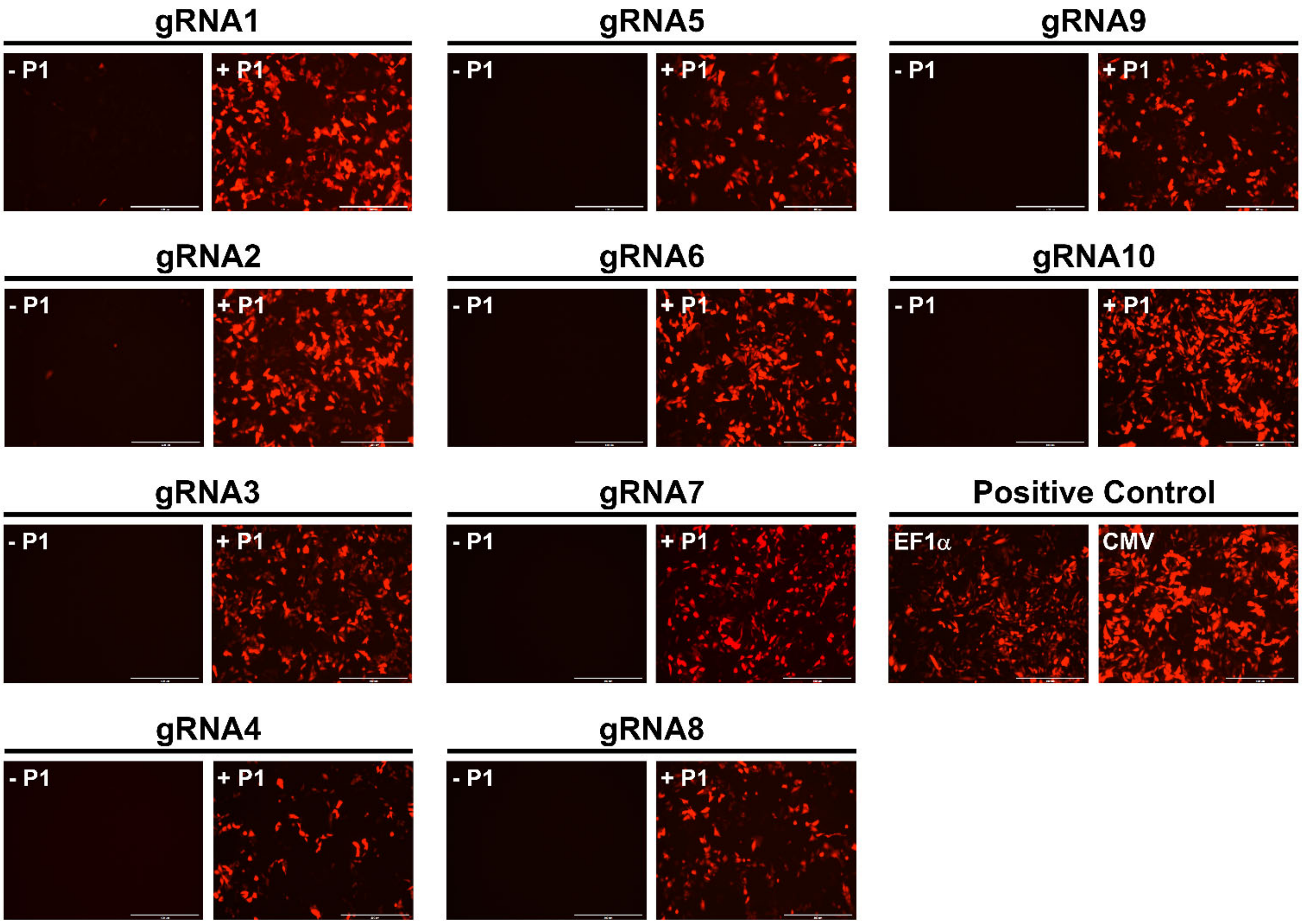

Supplementary Fig. 1 Comparison of episomal gene expression levels with ten distinct gRNA sequences. Each gRNA was paired with a corresponding synthetic operator containing 8x gRNA BS to control mKate transcription. CHO-K1 cells were transiently transfected, as illustrated in Fig. 1c, with gRNA constitutively expressed by the U6 promoter from plasmid #1 (P1). Experimental groups were transfected with all four plasmids, including P1 (+ P1); negative control groups (no gRNA) were transfected without P1 (- P1). Plasmids with mKate expression driven by constitutive promoters (EF1α or CMV), transfected at the same concentration, served as positive controls. Positive and negative control groups were also supplemented with a dummy plasmid to ensure that all groups had the same total amount of transfected plasmids. Representative fluorescent images revealed a wide range of mKate expression among the ten different gRNAs at 48 hours post-transfection. Only gRNA1 and gRNA2 exhibited slight leakage of mKate expression without the presence of P1. The results were independently repeated two times to confirm the reproducibility. Scale bars: 400 µm.

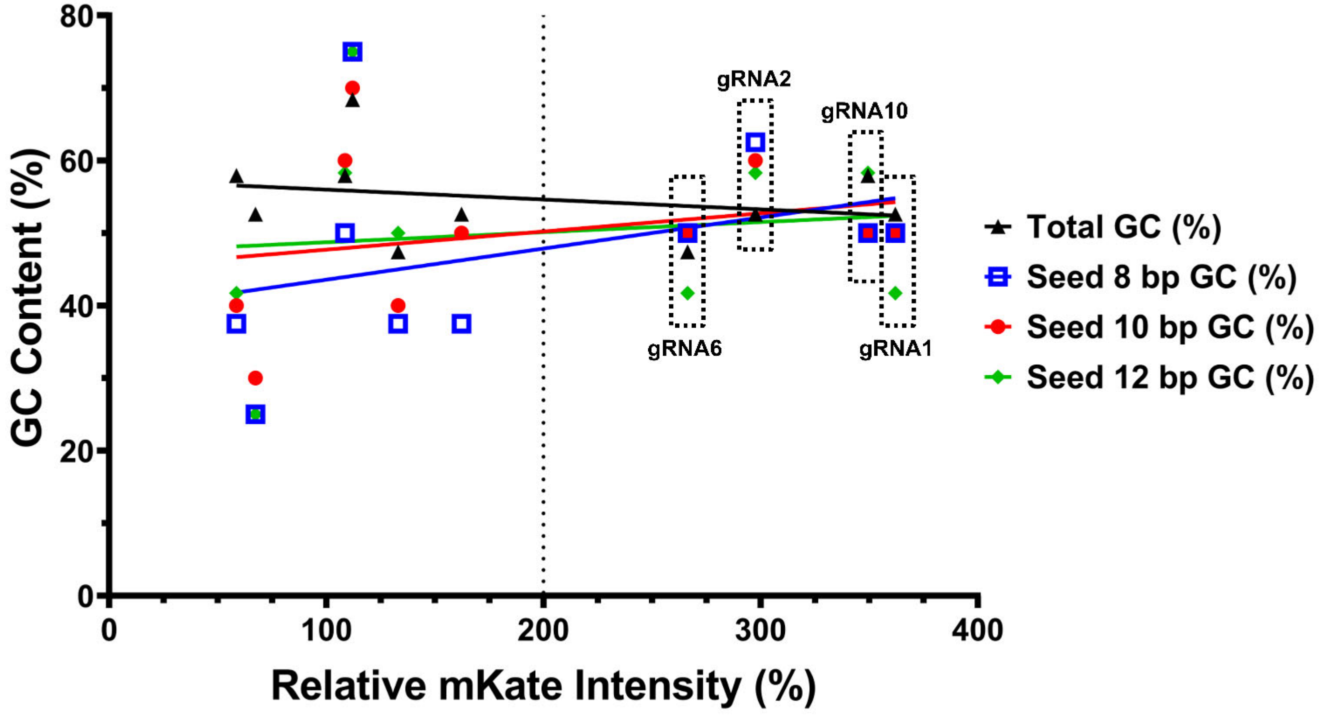

Supplementary Fig. 2 Analysis of the correlation between GC content in gRNA seed sequences and gene expression levels. We analyzed the relationship between GC content in gRNA seed 8, 10, and 12 bp sequences and corresponding mKate expression levels among gRNA1-10. Overall, gRNA1, 2, 6, and 10, which exhibited high mKate expression levels (>200% of EF1α promoter), all had 50-60% GC content in gRNA seed sequences, especially within seed 8-10 bp. Source data are provided as a Source Data file.

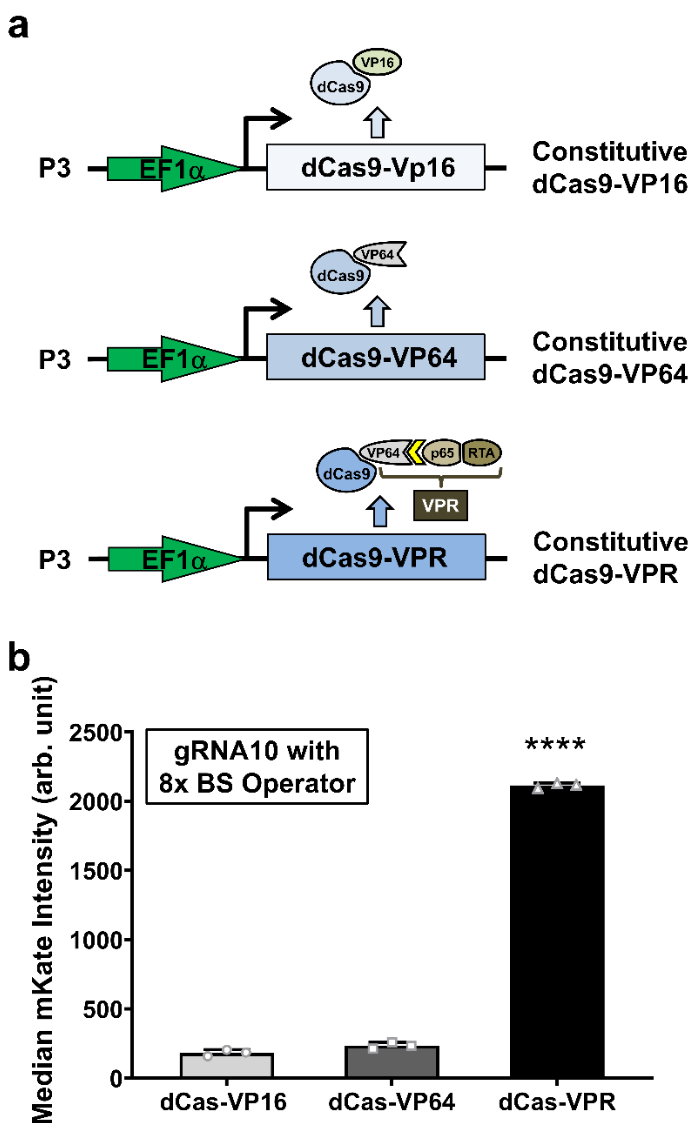

Supplementary Fig. 3 Comparison of gene expression levels obtained with three crisprTFs. mKate expression levels were compared for three crisprTFs: dCas9-VP16, dCas9-VP64, and dCas9-VPR, with gRNA10 (P1) and its 8x BS synthetic operator (P2) as depicted in Fig. 1c. **a** Schematic illustration of three versions of plasmid #3 (P3) respectively encoding the three crisprTFs composed of deactivated SpCas9 (dCas9) and transcriptional activation domains (TADs): dCas9-VP16, dCas9-VP64, and dCas9-VPR. **b** FACS results showed that dCas9-VPR had markedly higher median mKate expression than dCas9-VP16 (11.5 fold, $p<0.0001$) and dCas9-VP64 (9 fold, $p<0.0001$). Data were presented as median mKate intensity of the entire transfected population with arbitrary units (arb. unit). Data represent the mean ± SD (n = 3) (one-way ANOVA with multiple comparisons corrected by Dunnett test; ****$p<0.0001$). Source data are provided as a Source Data file.

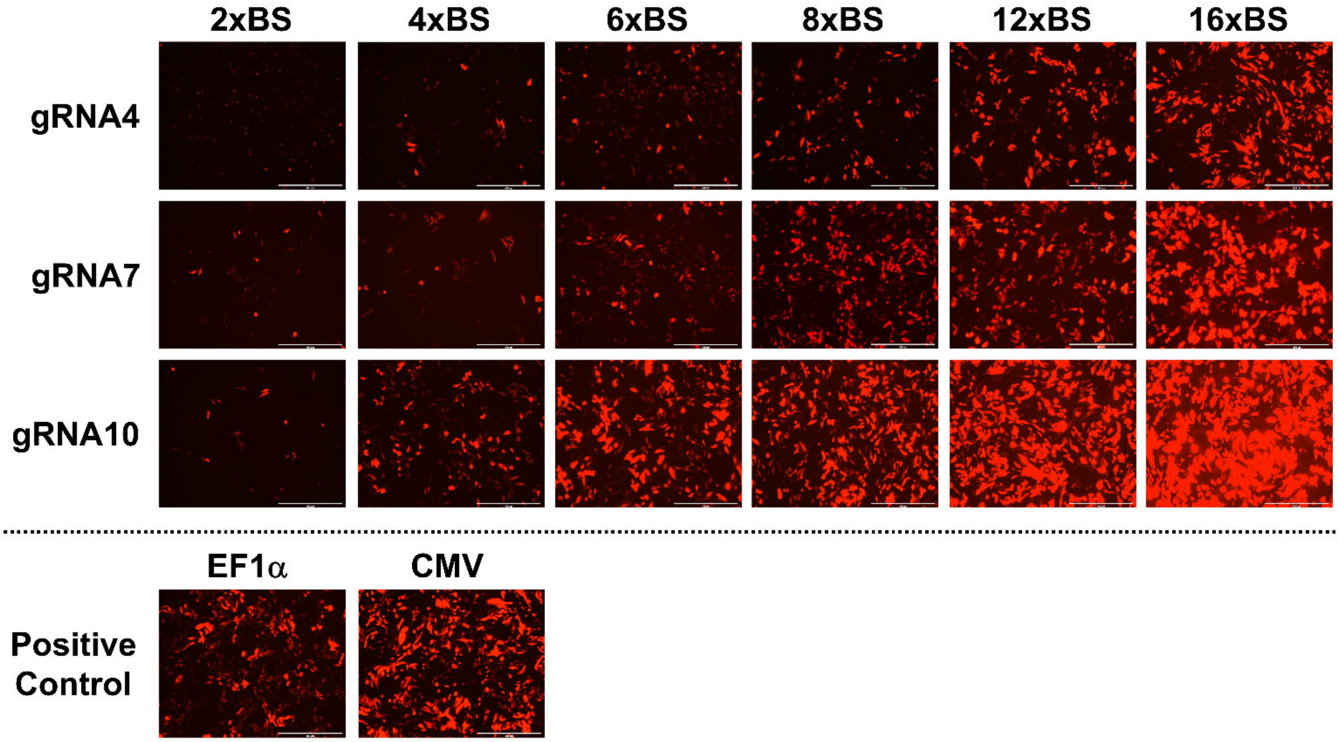

Supplementary Fig. 4 Comparison of gene expression levels with six distinct synthetic operators containing different numbers of gRNA BS in three gRNA series. CHO-K1 cells were transfected, as illustrated in Fig. 1c, with each gRNA constitutively expressed by the U6 promoter from Plasmid #1 (P1) and mKate expressed by each synthetic operator from Plasmid #2 (P2). mKate expression driven by EF1α and CMV promoters served as positive controls. Representative fluorescent images showed a dramatic range of mKate expression among six synthetic operators in all three gRNA series at 48 hours post-transfection, especially in the gRNA10 series. Results were independently repeated at least two times to confirm the reproducibility. Scale bars: 400 μm.

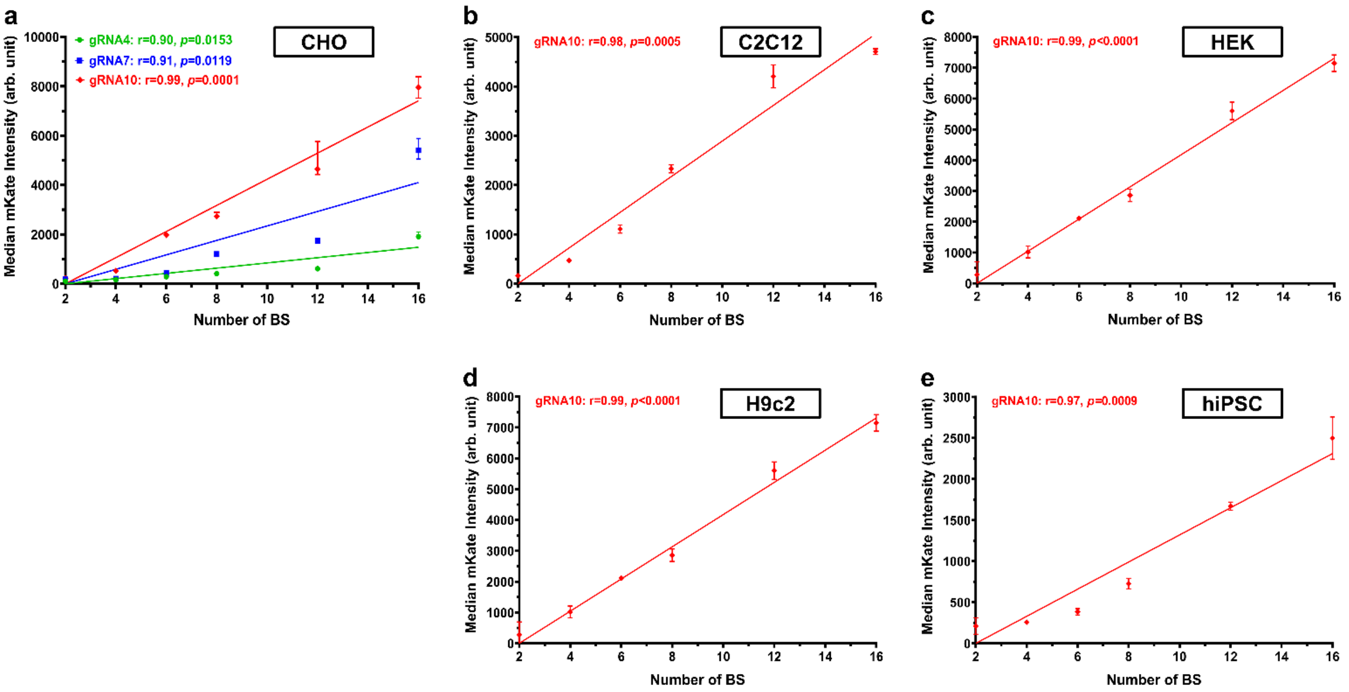

Supplementary Fig. 5 Correlation between the number of gRNA BS in the synthetic operator and the gene expression level. Based on the quantitative results from flow cytometry analyses presented in Fig. 2, we performed a Pearson correlation analysis to reveal the relationship between the number of gRNA BS in each synthetic operator of the three gRNA series and its target gene expression level. **a** In CHO-K1 cells, the gRNA4 series had the Pearson correlation coefficient (r)=0.90 (R^2=0.80, *p*=0.0153); the gRNA7 series had r=0.91 (R^2=0.83, *p*=0.0119); and the gRNA10 series had r=0.99 (R^2=0.98, *p*=0.0001). (**b**-**e**) For the gRNA10 series, r=0.98 (R^2=0.96, *p*=0.0005) in mouse C2C12 myoblasts (**b**), r=0.99 (R^2=0.99, *p*<0.0001) in human HEK293T cells (**c**), r=0.99 (R^2=0.99, *p*<0.0001) in rat H9C2 cardiomyoblast cells (**d**), and r=0.97 (R^2=0.95, *p*=0.0009) in hiPSC cells (**e**). Simple linear regression was performed to plot the graphs. All data were presented as median mKate intensity of the entire transfected population with arbitrary units (arb. unit). Data represent the mean ± SD (n = 3). Source data are provided as Source Data files.

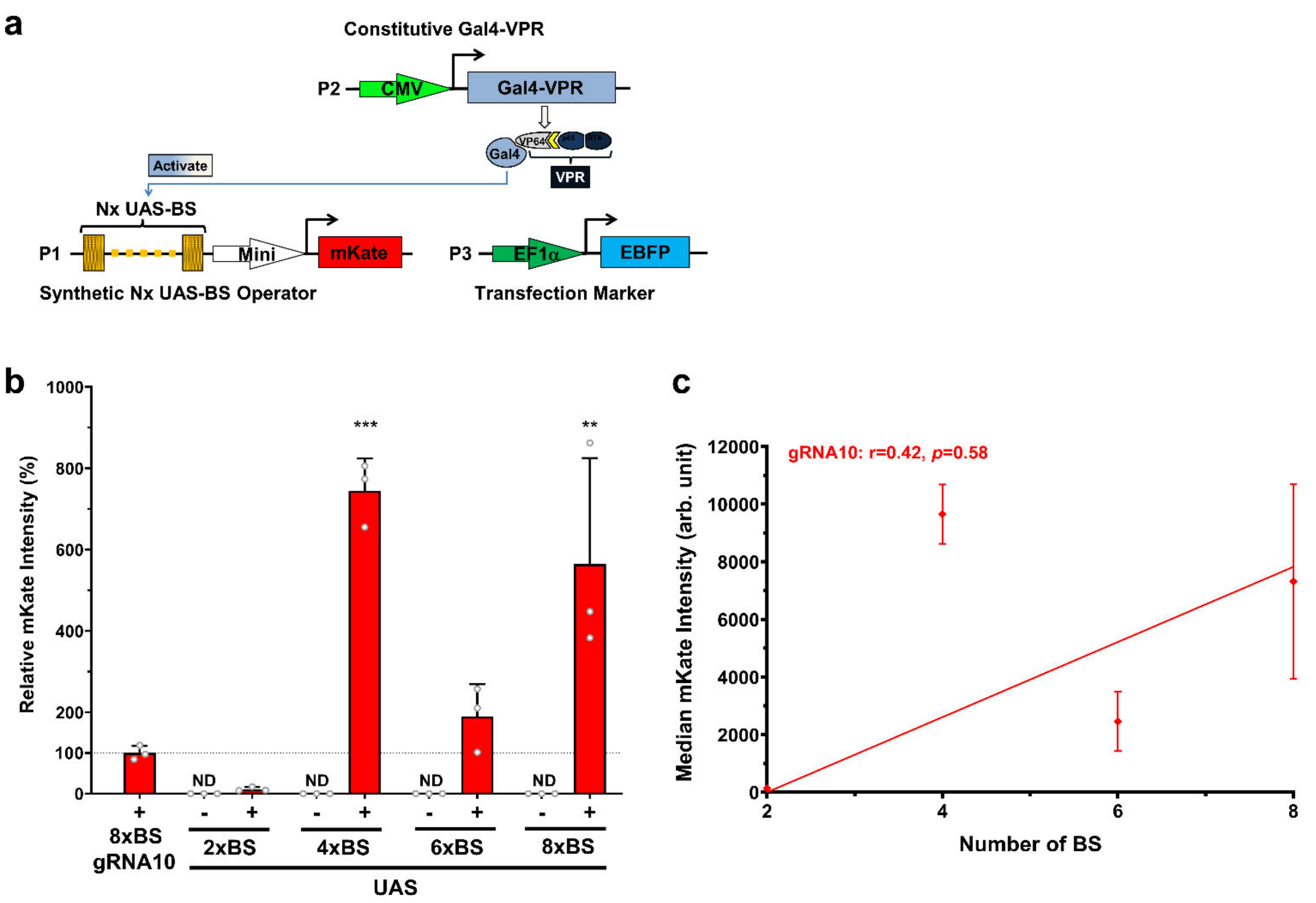

Supplementary Fig. 6 Gene expression controlled by Gal4-VPR/UAS system in CHO-K1 cells. a A schematic illustration of three plasmids used to transiently transfect CHO-K1 cells: plasmid #1 (P1) encoding the synthetic operator with 2-8x of UAS-BS to drive mKate expression; plasmid #2 (P2) constitutively expressing a Gal4-VPR gene; and plasmid #3 (P3) constitutively expressing the transfection marker (EBFP). **b** The mKate signal intensities of representative circuits with 2x-8x UAS-BS, relative to a landmark crisprTF promoter with 8x gRNA10-BS. Experimental groups (+), represented by solid red bars, were transfected with three plasmids (P1-P3). Control groups (-), represented by paired red hollow bars, were transfected without P2 (only P1 and P3) to detect baseline UAS operator leakage (UAS 4x BS and 8x BS vs. gRNA10 8x BS: p=0.0004 and p=0.0040, respectively). **c** Correlation between the number of UAS-BS in the synthetic operator and the gene expression level. Pearson correlation coefficient (r)=0.42 (R^2=0.18, p=0.58). Data were presented as median mKate intensity of the entire transfected population with arbitrary units (arb. unit). All data represent the mean ± SD (n = 3) (one-way ANOVA with multiple comparisons corrected by Dunnett test; **p<0.01, ***p<0.001; ND: not detected). Source data are provided as Source Data files.

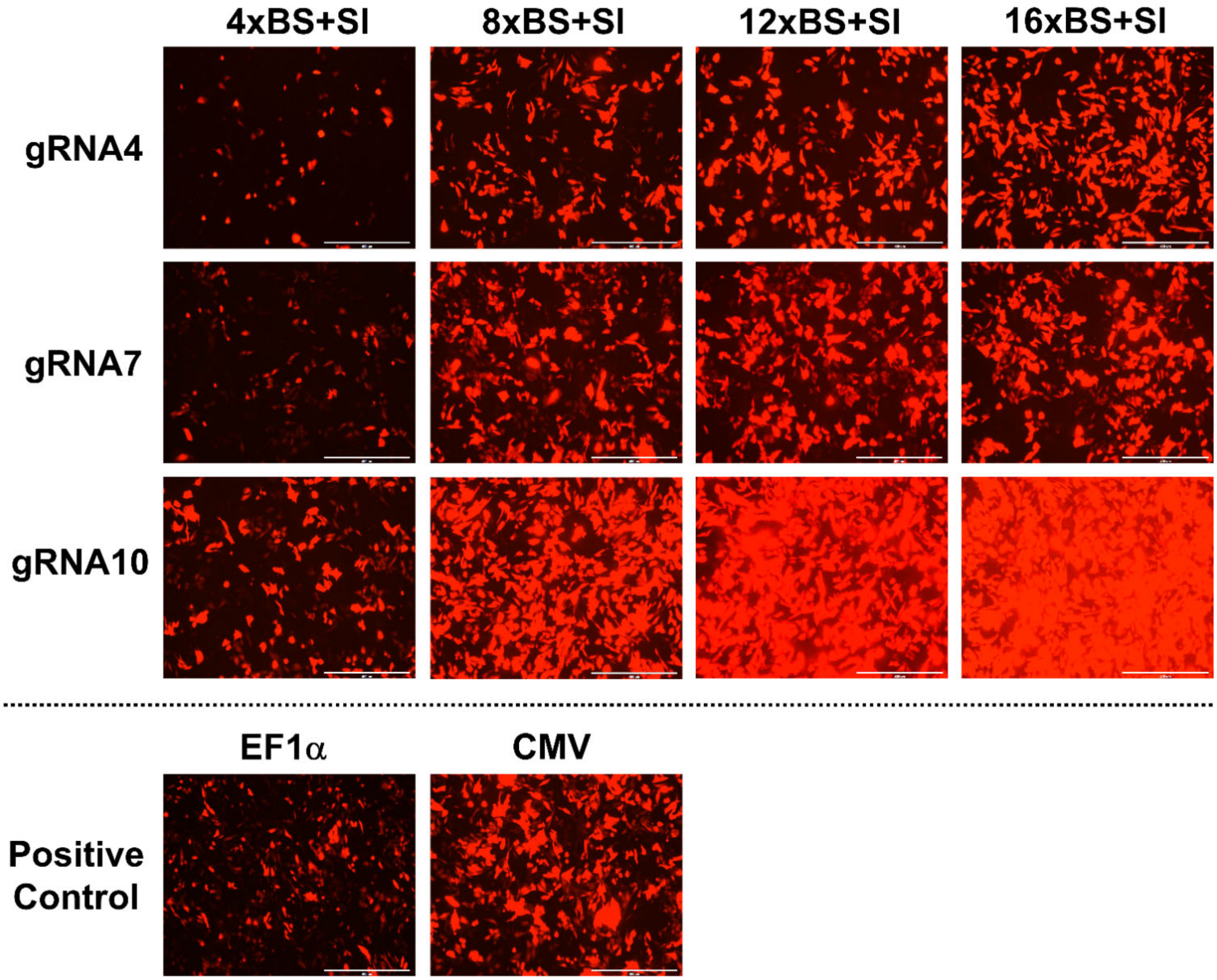

Supplementary Fig. 7 Comparison of gene expression levels with the addition of a synthetic intron (SI) in four distinct synthetic operators in three gRNA series. CHO-K1 cells were transfected, as illustrated in Fig. 1c and 3d, with each gRNA expressed constitutively from P1. mKate was expressed by each synthetic operator (P2) with the presence of an SI at the 5' UTR of the mKate gene. mKate expression driven by EF1α or CMV promoters served as positive controls. Representative fluorescent images revealed marked increases in mKate expression with the addition of the SI in 4 different synthetic operators of each gRNA series at 48 hours post-transfection. The increment was particularly noteworthy in the gRNA10 series when compared with mKate signals expressed by the same synthetic operators without the SI (data shown in Supplementary Fig. 4). These results were independently repeated three times to confirm the reproducibility. Scale bars: 400 μm.

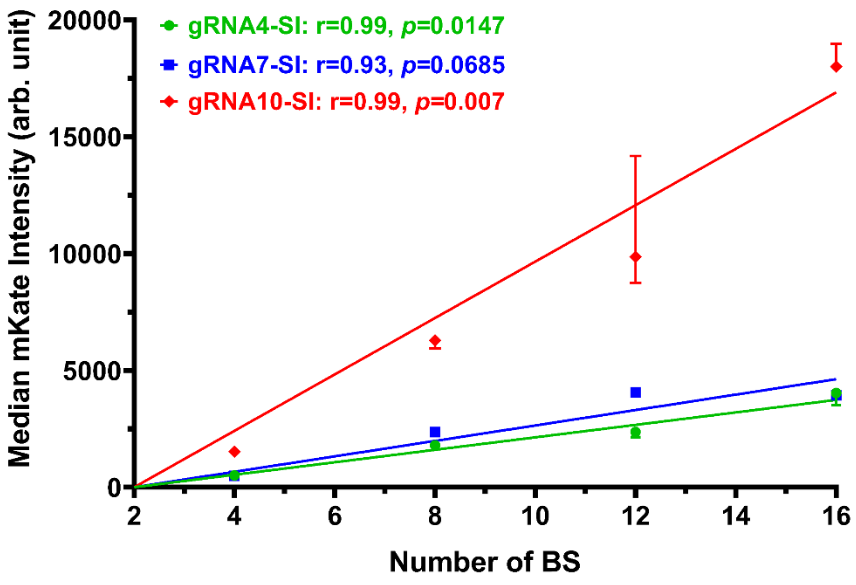

Supplementary Fig. 8 Correlation between the number of gRNA BS and the gene expression level with the addition of a synthetic intron (SI). Pearson correlation analysis uncovered the relationship between the number of gRNA BS in the synthetic operator and the associated gene expression level with the presence of an SI at the 5' UTR of the target gene. The gRNA4-SI series had the Pearson correlation coefficient (r)=0.99 (R^2=0.97, *p*=0.0147); the gRNA7-SI series had r=0.93 (R^2=0.87, *p*=0.0685); and the gRNA10-SI series had r=0.99 (R^2=0.99, *p*=0.007). Simple linear regression was performed to plot the graph. Data were presented as median mKate intensity of the entire transfected population with arbitrary units (arb. unit). Data represent the mean ± SD (n = 3). Source data are provided as a Source Data file.

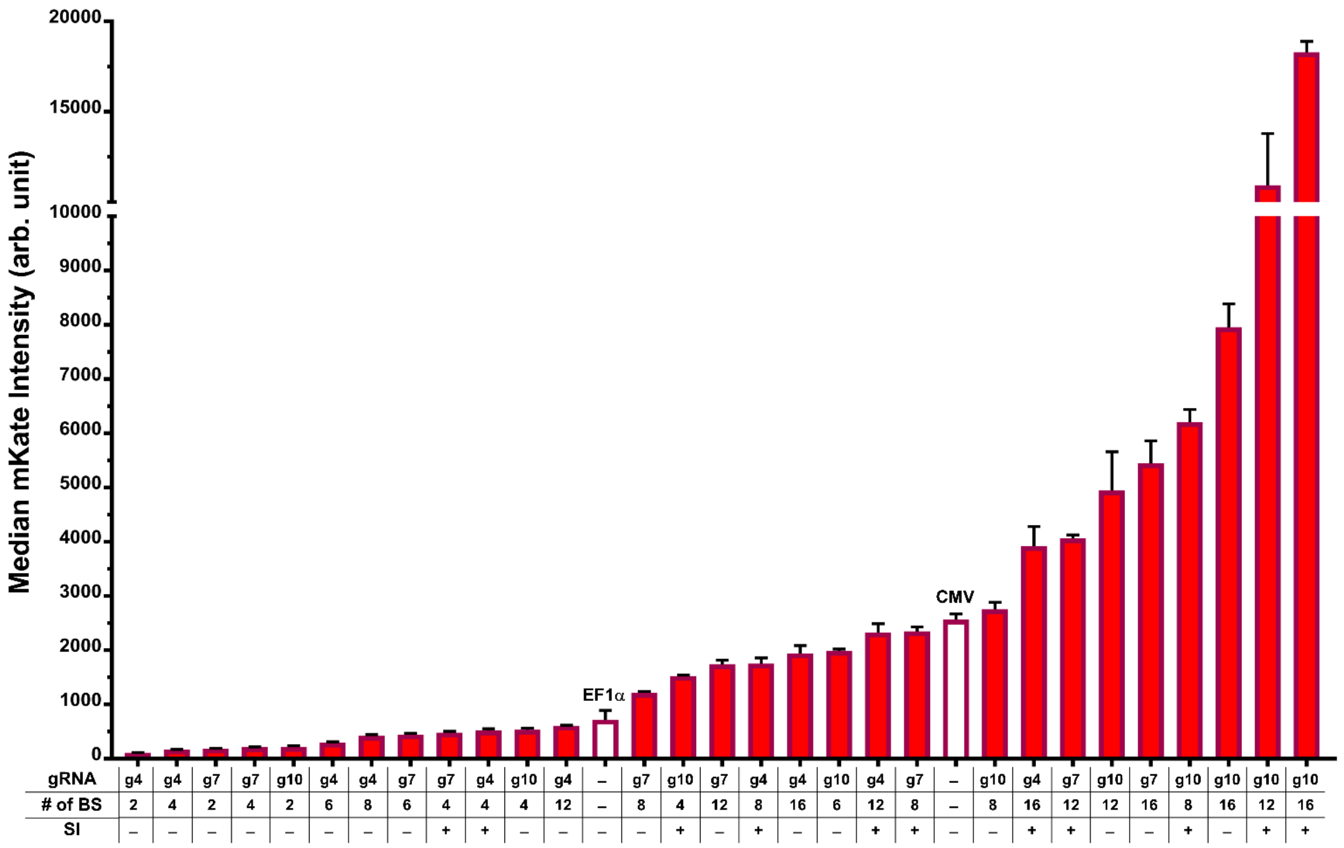

Supplementary Fig. 9 Summary of construct compositions and corresponding gene expression levels. Compositions and unnormalized gene expression levels of constructs from gRNA4 (g4), gRNA7 (g7), and gRNA10 (g10) series that were tested episomally in CHO cells, with or without the synthetic intron (SI), in Fig. 2a and 3d. EF1α and CMV promoter controls are represented by empty bars. Data represent the group mean ± SD (n = 3). All original data points can be found in Fig. 2a and 3d. Source data are provided as a Source Data file.

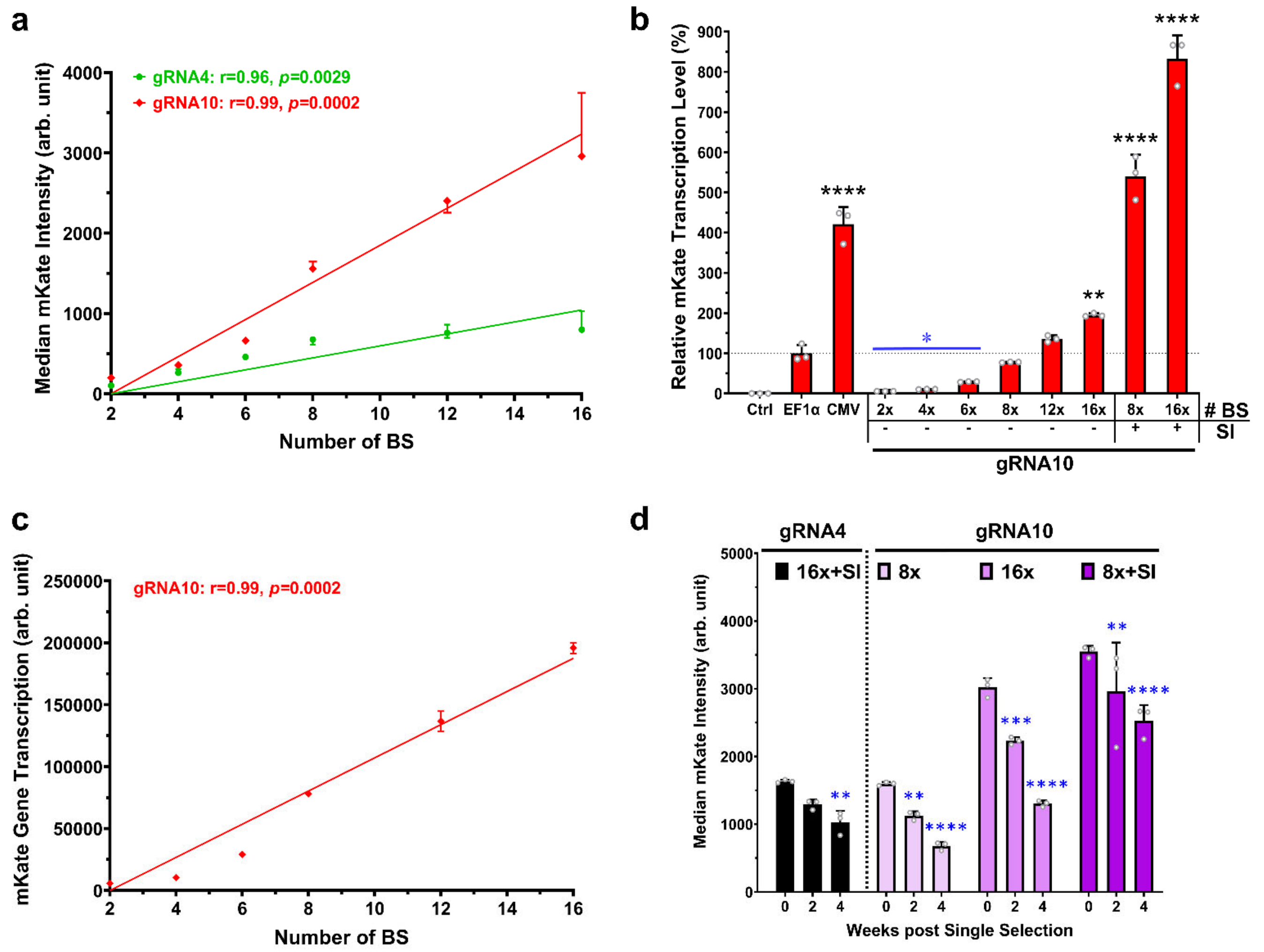

Supplementary Fig. 10 Analyses of chromosomally integrated crisprTF promoter circuits in sLP-CHO cells. a Pearson correlation analysis showed the relationship between the number of gRNA BS in the synthetic operator and the associated gene expression level when gene circuits were chromosomally integrated. The gRNA4 series had the Pearson correlation coefficient (r)=0.96 (R^2=0.91, *p*=0.0029), and the gRNA10 series had r=0.99 (R^2=0.98, *p*=0.0002). Simple linear regression was performed to plot the graph. **b** RT-qPCR analysis showing mKate transcription of chromosomally integrated crisprTF promoter circuits relative to the integrated EF1α control (one-way ANOVA with multiple comparisons corrected by Dunnett test; CMV and gRNA10 2x, 4x, 6x and 16x BS without SI vs. EF1α: *p*<0.0001, *p*=0.0038, *p*=0.0061, *p*=0.0379, and *p*=0.0035, respectively; gRNA10 8x and 16x BS with SI vs. EF1α: *p*<0.0001 and *p*<0.0001, respectively). Data represent the mean ± SD (n = 3); for increased expression (marked in black): ***p*<0.01; *****p*<0.0001; for decreased expression (marked in blue): **p*<0.05. **c** Correlation analysis between the number of gRNA10 BS and mKate transcription levels showed r=0.99 (R^2=0.98, *p*=0.0002). **d** The mKate expression levels of representative circuit integrants from gRNA4 (16x BS with SI) and gRNA10 (8x and 16x BS without SI, and 8x BS with SI) series over the course of 4 weeks following single antibiotic selection (gRNA4 16x BS with SI: 0 vs. 4 weeks: *p*=0.0014; gRNA10 8x BS without SI: 0 vs. 2 weeks: *p*=0.0095, 0 vs. 4 weeks: *p*<0.0001; gRNA10 16x BS without SI: 0 vs. 2 weeks: *p*=0.0001, 0 vs. 4 weeks: *p*<0.0001; gRNA10 8x BS with SI: 0 vs. 2 weeks: *p*=0.0018, 0 vs. 4 weeks: *p*<0.0001). Data represent the mean ± SD (n = 3) (two-way ANOVA with multiple comparisons corrected by Dunnett test, Time x BS: *p*=0.0034, Time: *p*<0.0001, BS: *p*<0.0001; for decreased expression marked in blue: ***p*<0.01, ****p*<0.001, *****p*<0.0001). Source data are provided as Source Data files.

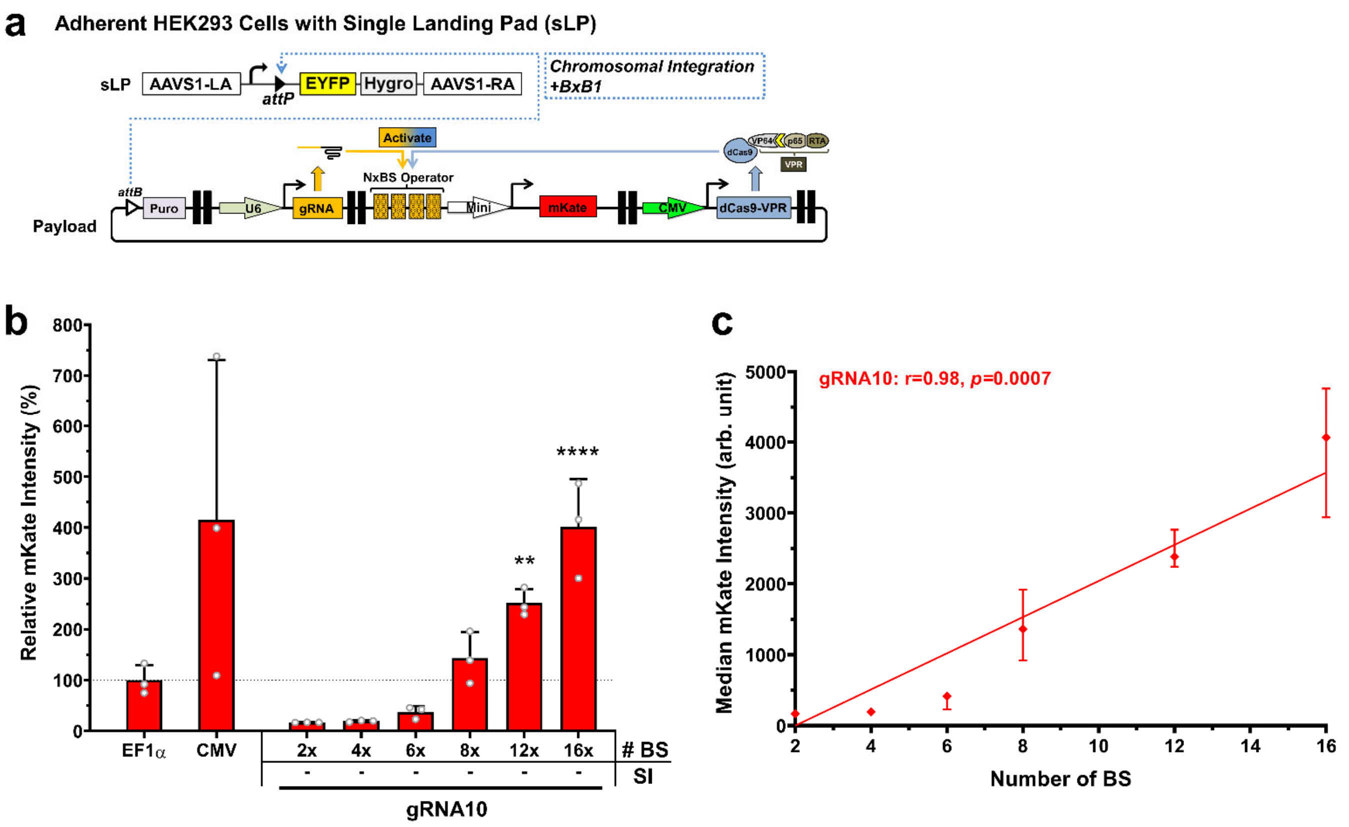

Supplementary Fig. 11 Genomic integration and precision gene expression in HEK293 landing pad cells. a A schematic illustration of an integration gene circuit and BxB1 recombinase-mediated, site-specific integration in an engineered, adherent HEK293 cell line with a single landing pad (sLP). Positive integration control circuits had a central TU with an EF1α or CMV promoter driving mKate expression and two flanking dummy TUs with no gene expression in the same architecture. **b** The mKate signal intensities of the chromosomally integrated payload circuits in sLP-HEK293 cells relative to the integrated EF1α control circuit at 1-week post-selection (gRNA10 12x and 16x BS vs. EF1α: p=0.0038 and p<0.0001, respectively). **c** Correlation between the number of gRNA10 BS and mKate expression levels. Data were presented as median mKate intensity of the entire transfected population with arbitrary units (arb. unit). Pearson correlation coefficient (r)=0.98 (R^2=0.96, p=0.0007). All data represent the mean ± SD (n = 3) (one-way ANOVA with multiple comparisons corrected by Dunnett test; **p<0.01, ****p<0.0001). Source data are provided as Source Data files.

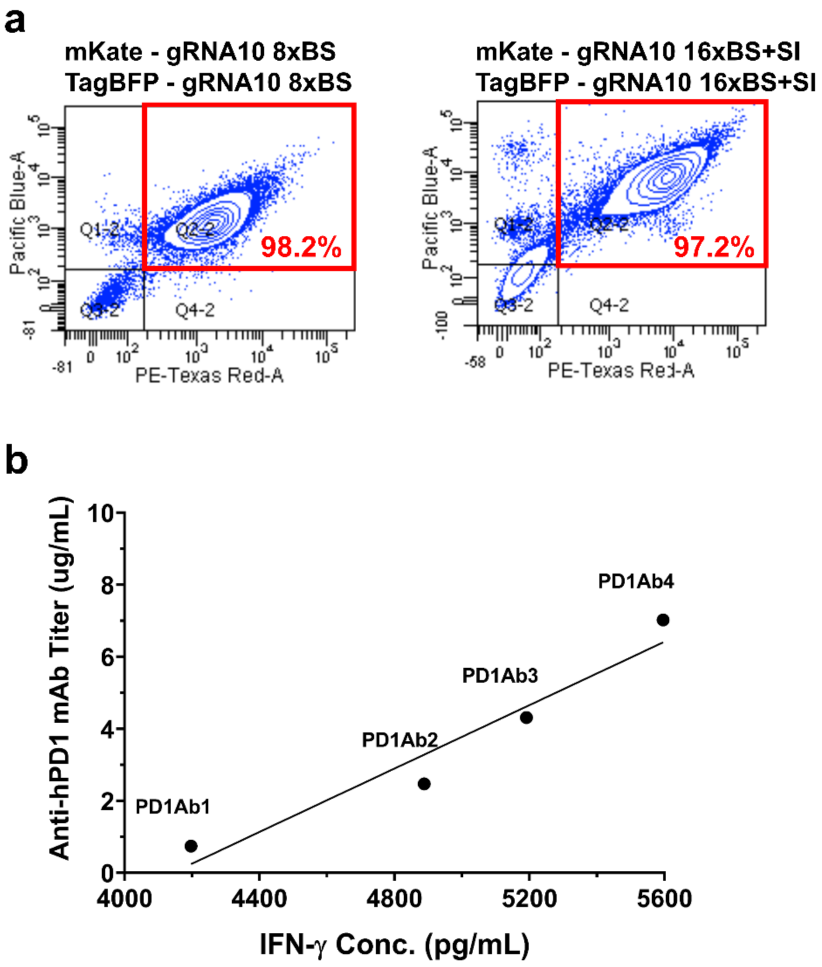

Supplementary Fig. 12 Genomic integration and precision control of target gene expression in the CHO cells engineered with a double landing pad (dLP). a Precision control of expression levels of two target genes in integrated dLP-CHO cells. Representative FACS dot-plots showing the mKate (x-axis, PE-Texas Red) and TagBFP (y-axis, Pacific Blue) signals in dLP-CHO cells integrated with either 8x BS without SI (left panel) or 16x BS with SI (right panel) control circuit**. b** Correlation between IFN-γ production and the anti-hPD1 titer before the start of co-culturing. Pearson correlation analysis revealed the relationship between IFN-γ production and the anti-hPD1 titer in the dLP-CHO cell cultures pre-seeded for two days prior to the start of co-culturing (n=3). Pearson correlation coefficient (r)=0.97 (R^2=0.94, *p*=0.0327). Source data are provided as a Source Data file.

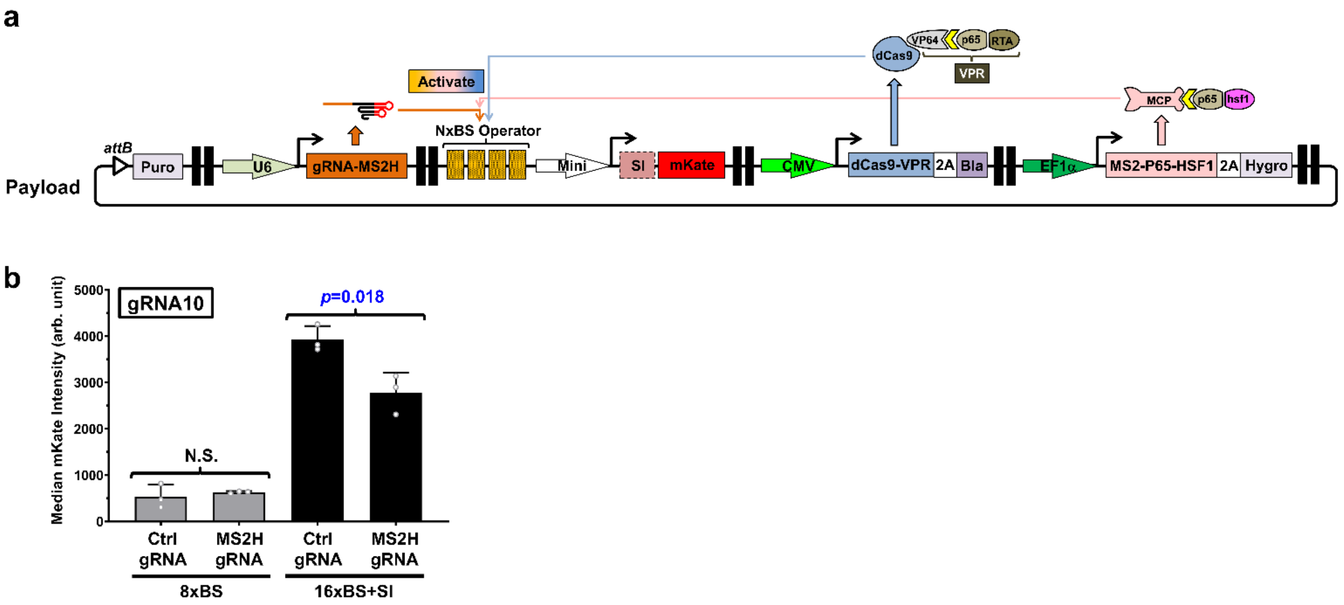

Supplementary Fig. 13 Investigation of the synergistic effect between SAM and dCas9-VPR at the chromosomal level. a A schematic illustration of the gene circuit for chromosomal integration in sLP-CHO cells. The circuit constitutively co-expressed SAM, including gRNA with hairpins and MCP-p65-hsf1, and dCas9-VPR as well as three selection markers, including the 5' flanking puromycin and the 3' flanking blasticidin (associated with the *dCas9-VPR* gene using a self-cleaving *P2A* peptide sequence) and hygromycin (associated with *MS2-P65-HSF1* gene using a *P2A sequence*). **b** sLP-CHO cells were transfected with the payload circuit and a BxB1-expressing plasmid. After the triple-antibiotic selection, mKate expression levels assessed by flow cytometry showed no synergistic effect between SAM and dCas9-VPR with the chromosomally integrated gRNA10 8xBS operator (p>0.05). mKate signals significantly decreased when SAM and dCas9-VPR were acting together with the gRNA10 16xBS with SI operator (p=0.018). Data were presented as median mKate intensity of the entire transfected population with arbitrary units (arb. unit). Data represent the mean ± SD (n = 3) (two-tailed paired Student's t-test). Source data are provided as a Source Data file.

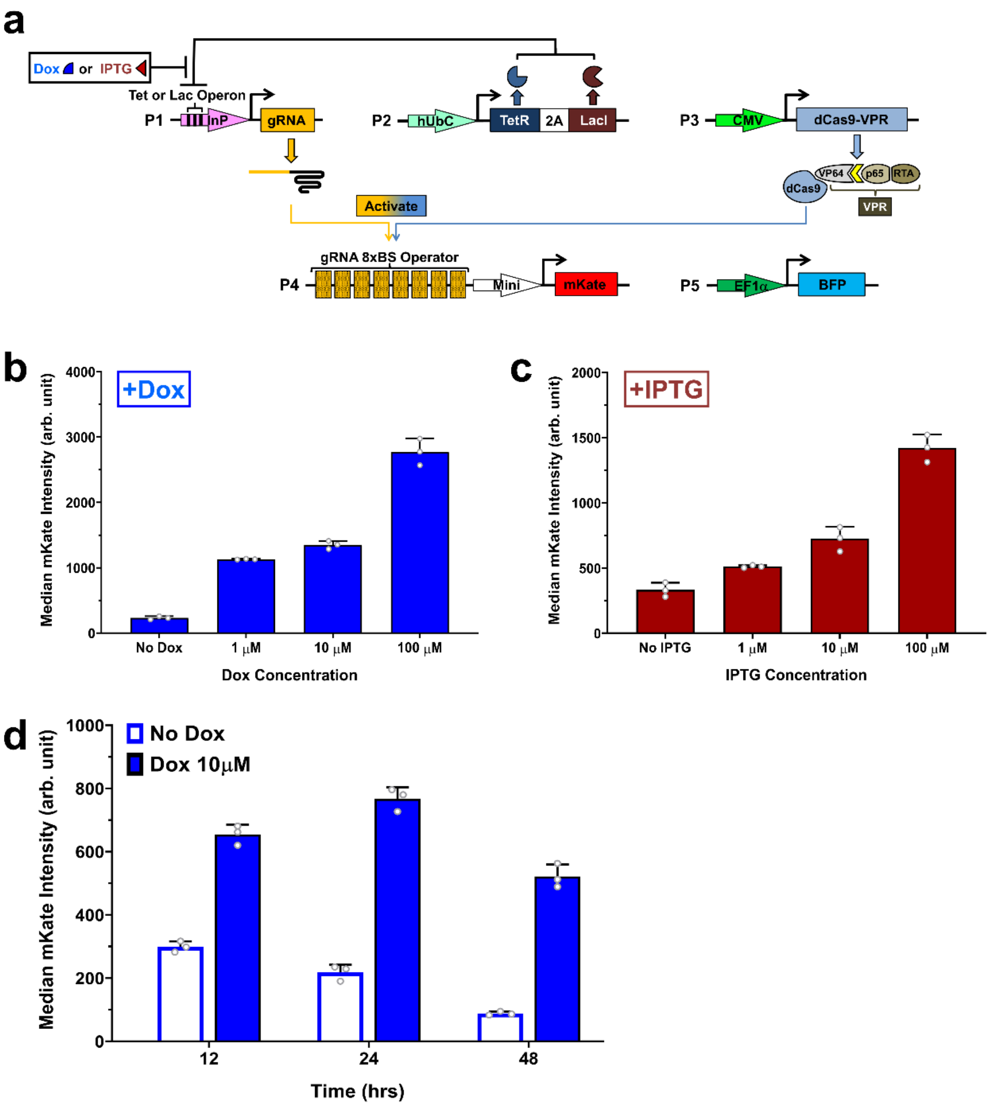

Supplementary Fig. 14 crisprTF promoters with small molecule-inducible gRNA expression. a To equip the crisprTF promoter system with an added tier of controllability, we developed inducible switches with doxycycline (Dox)-inducible or isopropyl β-D-1-thiogalactopyranoside (IPTG)-inducible gRNA expression. Either a Tet or a Lac operon was custom embedded into an RNA polymerase III (Pol III) promoter driving gRNA10 expression to render inducibility. Without an appropriate small molecule inducer (Dox or IPTG), the Tet repressor or Lac inhibitor (both constitutively expressed from a single plasmid using a self-cleaving P2A peptide) bound to the Tet or Lac operon, respectively, and repressed gRNA expression. In the presence of Dox or IPTG, the Tet repressor or Lac inhibitor did not bind to the respective operon, permitting gRNA10 transcription. **b** Titration analysis of the Dox-inducible gRNA10 expression with its 8x BS synthetic promoter unveiled incrementally increased mKate expression with increased Dox concentration; the highest expression level was seen with 100 μM Dox. **c** Titration analysis of the IPTG-inducible gRNA10 expression with its 8x BS synthetic promoter revealed incrementally increased mKate expression with increased IPTG concentration; the highest expression level was seen with 100 μM IPTG. **d** mKate expression kinetics in the presence (solid blue bars) or absence (empty blue bars) of 10 μM Dox. Data were presented as median mKate intensity of the entire transfected population with arbitrary units (arb. unit). All data represent the mean ± SD (n = 3). Source data are provided as Source Data files.

Supplementary Table 1. Sequences of key genetic parts and primers

Genetic Part Name	DNA sequence	Appearance in Figure(s)
2A sequence	ggatctggcgccaccaacttctctctgctgaagcaggccggcgacgtggaggagaacccaggcccc	4, 5, 6, S13, S14
2x cHS4 insulator	aagctttttccccgtatccccccaggtgtctgcaggctcaaagagcagcgagaagcgttcagaggaaagcgatcccgtgccaccttccccgtgcccgggctgtccccgcacgctgccggctcggggatgcgggg ggagcgccggaccggagcggagccccgggcggctcgctgctgccccctagcgggggagggacgtaattacatccctgggggctttgggagggggctgtccccgtgagctcccaggcgcgcctgtcattctaaatctctctttcagcctaaagctttttccccgtatccccccaggtgtctgcaggctcaaagagcagcgagaagcgttcagaggaaagcgatcccgtgccaccttccccgtgcccgggctgtccccgcacgctgccggctcggggatgcgggggagcgccggaccggagcggagccccgggcggctcgctgctgccccctagcgggggagggacgtaattacatccctgggggctttgggggggggctgtccccgtgagctc	1, 4, 5, 6, S11, S13
gRNA scaffold	NNNNNNNNNNNNNNNNNNNNgttttagagctagaaatagcaagttaaaataaggctagtccgttatcaacttgaaaaagtggcaccgagtcggtgc	1-6, S1-S14
sgRNA10 16x BS + mini CMV promoter	tacgagggcgatccaacgactggtacgagggcgatccaacgacgggtacgagggcgatccaacgacaggtacgagggcgatccaacgaccggtacgagggcgatccaacgactggtacgagggcgatccaacgacgggtacgagggcgatccaacgacaggtacgagggcgatccaacgaccggtacgagggcgatccaacgactggtacgagggcgatccaacgacgggtacgagggcgatccaacgacaggtacgagggcgatccaacgaccggtacgagggcgatccaacgactggtacgagggcgatccaacgacgggtacgagggcgatccaacgacaggtacgagggcgatccaacgaccggtggtctatataagcagagctcgtttagtgaaccgtcagatcgcctggagacgccatccacgctgttttgacctccatagaagac	2-6, S4-S14
sgRNA10 16x BS + mini CMV promoter + synthetic intron	tacgagggcgatccaacgactggtacgagggcgatccaacgacgggtacgagggcgatccaacgacaggtacgagggcgatccaacgaccggtacgagggcgatccaacgactggtacgagggcgatccaacgacgggtacgagggcgatccaacgacaggtacgagggcgatccaacgaccggtacgagggcgatccaacgactggtacgagggcgatccaacgacgggtacgagggcgatccaacgacaggtacgagggcgatccaacgaccggtacgagggcgatccaacgactggtacgagggcgatccaacgacgggtacgagggcgatccaacgacaggtacgagggcgatccaacgaccggtggtctatataagcagagctcgtttagtgaaccgtcagatcgcctggagacgccatccacgctgttttgacctccatagaagacgtgagtactccctctcaaaagcgggcatgacttctgcgctaagattgtcagtttccaaaaacgaggaggatttgatattcacctggcccgcggtgatgcctttgagggtggccgcgtccatctggtcagaaaagacaatctttttgttgtcaagcttgaggtgtggcaggcttgagatctggccatacacttgagtgacaatgacatccactttgcctttctctccacag	3-6, S7-S10, S12-S13
sgRNA4 16x BS + mini CMV promoter	cgcgagggtagttgacaacgtggcgcgagggtagttgacaacggggcgcgagggtagttgacaacgaggcgcgagggtagttgacaacgcggcgcgagggtagttgacaacgtggcgcgagggtagttgacaacggggcgcgagggtagttgacaacgaggcgcgagggtagttgacaacgcggcgcgagggtagttgacaacgtggcgcgagggtagttgacaacggggcgcgagggtagttgacaacgaggcgcgagggtagttgacaacgcggcgcgagggtagttgacaacgtggcgcgagggtagttgacaacggggcgcgagggtagttgacaacgaggcgcgagggtagttgacaacgcggtggtctatataagcagagctcgtttagtgaaccgtcagatcgcctggagacgccatccacgctgttttgacctccatagaagac	2, 3, 4, S4, S5, S7-S10
sgRNA7 16x BS + mini CMV promoter	agtacgggtcgtacacgaagtggagtacgggtcgtacacgaaggggagtacgggtcgtacacgaagaggagtacgggtcgtacacgaagcggagtacgggtcgtacacgaagtggagtacgggtcgtacacgaaggggagtacgggtcgtacacgaagaggagtacgggtcgtacacgaagcggagtacgggtcgtacacgaagtggagtacgggtcgtacacgaaggggagtacgggtcgtacacgaagaggagtacgggtcgtacacgaagcggagtacgggtcgtacacgaagtggagtacgggtcgtacacgaaggggagtacgggtcgtacacgaagaggagtacgggtcgtacacgaagcggtggtctatataagcagagctcgtttagtgaaccgtcagatcgcctggagacgccatccacgctgttttgacctccatagaagac	2, 3, S4, S5, S7-S9
U6 promoter + gRNA10 + MS2 Stem Loop RNA	agagggcctatttcccatgattccttcatatttgcatatacgatacaaggctgttagagagataattagaattaatttgactgtaaacacaaagatattagtacaaaatacgtgacgtagaaagtaataatttcttgggtagtttgcagttttaaaattatgttttaaaatggactatcatatgcttaccgtaacttgaaagtatttcgatttcttggctttatatatcttgtggaaaggacgaaacaccgtacgagggcgatccaacgagttttagagctaggccaacatgaggatcacccatgtctgcagggcctagcaagttaaaataaggctagtccgttatcaacttggccaacatgaggatcacccatgtctgcagggccaagtggcaccgagtcggtgctttttttctag	3, S13
VPR (VP64-SV40NLS-p65-RTA)	gaggccagcggttccggacgggctgacgcattggacgattttgatctggatatgctgggaagtgacgccctcgatgattttgaccttgacatgcttggttcggatgcccttgatgactttgacctcgacatgctcggcagtgacgcccttgatgatttcgacctggacatgctgattaactctagaagttccggatctccgaaaaagaaacgcaaagttggtagccagtacctgcccgacaccgacgaccggcaccggatcgaggaaaagcggaagcggacctacgagacattcaagagcatcatgaagaagtccccttcagcggccccaccgaccctagacctccacctagaagaatcgccgtgcccagcagatccagcgccagcgtgccaaaacctgccccccagccttaccccttcaccagcagcctgagcaccatcaactacgacgagttccctaccatggtgttccccagcggccagatctctcaggcctctgctctggctccagcccctcctcaggtgctgcctcaggctcctgctcctgcaccagctccagccatggtgtctgcactggctcaggcaccagcacccgtgcctgtgctggctcctggacctccacaggctgtggctccaccagcccctaaacctacacaggccggcgagggcacactgtctgaagctctgctgcagctgcagttcgacgacgaggatctgggagccctgctgggaaacagcaccgatcctgccgtgttcaccgacctggccagcgtggacaacagcgagttccagcagctgctgaaccagggcatccctgtggcccctcacaccaccgagcccatgctgatggaataccccgaggccatcacccggctcgtgacaggcgctcagaggcctcctgatccagctcctgcccctctgggagcaccaggcctgcctaatggactgctgtctggcgacgaggacttcagctctatcgccgatatggatttctcagccttgctgggctctggcagcggcagccgggattccagggaagggatgtttttgccgaagcctgaggccggctccgctattagtgacgtgtttgagggccgcgaggtgtgccagccaaaacgaatccggccatttcatcctccaggaagtccatgggccaaccgcccactccccgccagcctcgcaccaacaccaaccggtccagtacatgagccagtcgggtcactgaccccggcaccagtccctcagccactggatccagcgcccgcagtgactcccgaggccagtcacctgttggaggatcccgatgaagagacgagccaggctgtcaaagcccttcgggagatggccgatactgtgattccccagaaggaagaggctgcaatctgtggccaaatggacctttcccatccgcccccaaggggccatctggatgagctgacaaccacacttgagtccatgaccgaggatctgaacctggactcacccctgaccccggaattgaacgagattctggataccttcctgaacgacgagtgcctcttgcatgccatgcatatcagcacaggactgtccatcttcgacacatctctgttttga	1-6, S1, S3-S5, S7-S12
G.Luc sp CDS Signal peptide + Anti-huPD1 (5C4) Light Chain	ggagttaaggtattgttcgcattgatttgcatagccgtggccgaagctgaaatagtgctgacccagtcccccgccaccctctctctctcacccggagagagggcaactctgtcctgcagggcctcccaatccgtaagcagctatctggcttggtaccagcaaaaacccggacaggcaccccggctgttgatctatgacgcctccaaccgagccactggcatccccgccagattctccggatctggatcagggactgattttaccctgactatcagttctctggaacctgaagattttgcagtttattattgtcagcaaagctctaactggccaagaacctttggccagggcacgaaggtcgagataaaaactgtcgcggccccaagcgtctttatctttccgccgtctgatgaacagctgaaatcagggaccgcctctgtggtgtgtctgctgaacaacttctacccaagggaagctaaggtgcaatggaaagtagacaatgccttgcagagcggcaactctcaggagagcgtaaccgaacaggatagtaaggattcaacttacagtctgagctctaccctgactctgagtaaggccgattacgagaagcataaagtctacgcatgcgaggtcacacatcaaggtctttcatctcccgtgacaaagtcctttaatcgaggggaatgctaa	6

G.Luc sp Signal peptide + Anti-huPD1 (5C4) Heavy Chain	atgggagttaaggtattgttcgcattgatttgcatagccgtggccgaagctcaggtgcagctcgtagagagcgggggaggtgtcgttcagccagggaggagtctgcggctggactgcaaagcctccgggattactttc tcaaacagcgggatgcactgggtgaggcaggctcccggaaagggcctcgagtgggtggcggtaatatggtacgacgggagcaaacgctactacgcagacagtgtgaaaggaaggttcactatttctcgcgaca attcaaagaacaccctcttcctgcagatgaacagtctgcgggcagaagacaccgctgtctattactgtgccaccaatgacgattactggggccagggtaccctcgtgaccgtgtctagcgcctctaccaaaggacc atcagtttttcctcttgctccctgcagtcgctcaaccagcgagagcacagcagcgctgggatgtctggtgaaggattattttcctgagcccgtgactgtgagctggaattcaggcgccctgacgtccggcgtccatacatt cccagcggtacttcaaagtagcgggttgtactctctctctagcgtggtaaccgtaccgagctcctccctggggacgaaaacgtatacatgtaatgtcgatcacaaaccatctaacacaaaagtggacaaacgcgttg agtccaagtatggccctccatgcccaccctgccccgcaccggagtttctgggcgggcccagtgtctttctgttcccaccgaagcctaaggacacgttgatgatctcaagaacacctgaagtcacctgcgtagtcgtgg acgtttctcaggaggatcccgaggtccaattcaattggtacgttgatggagtggaggtccacaacgcaaagacaaagccgcgcgaagaacagttcaattctacttaccgcgttgttagcgtgctgactgtgctccacc aagactggctgaatggtaaggagtataagtgcaaggtgagcaataagggattgccatctagcatcgaaaagacaatatccaaagccaagggccaaccacgagagccacaagtgtacacgttgcctccctcac aagaagagatgaccaagaatcaagtgagcctcacttgcctggtcaagggattctacccttctgatatcgcagtggagtgggagtccaatggacagcccgagaacaactacaagacaacacccccagtgctggat tccgacggctcattcttcttgtatagccggctgacagtggacaagagcaggtggcaggaaggaaatgtcttctcctgctccgtgatgcacgaggccctccacaaccactacactcagaaatctctctctctttcactcgg taaataa	6
Gal4 DNA binding domain	atgaagctactgtcttctatcgaacaagcatgcgatatttgccgacttaaaaagctcaagtgctccaaagaaaaaccgaagtgcgccaagtgtctgaagaacaactgggagtgtcgctactctcccaaaaccaaaa ggtctccgctgactagggcacatctgacagaagtggaatcaaggctagaaagactggaacagctatttctactgatttttcctcgagaagaccttgacatgattttgaaaatggattctttacaggatataaaagcattgt taacaggattatttgtacaagataatgtgaataaagatgccgtcacagatagattggcttcagtggagactgatatgcctctaacattgagacagcatagaataagtgcgacatcatcatcggaagagagtagtaaca aaggtcaaagacagttgactgtatcg	S6
2x UAS for Gal4DBD binding + mini CMV promoter	cggagtactgtcctccgagcggagtactgtcctccgagtggtctatataagcagagctcgtttagtgaaccgtcagatcgcctggagacgccatccacgctgttttgacctccatagaagac	S6
4x UAS for Gal4DBD binding + mini CMV promoter	cggagtactgtcctccgagcggagtactgtcctccgagcggagtactgtcctccgagcggagtactgtcctccgagtggtctatataagcagagctcgtttagtgaaccgtcagatcgcctggagacgccatccacg ctgttttgacctccatagaagac	S6
6x UAS for Gal4DBD binding + mini CMV promoter	cggagtactgtcctccgagcggagtactgtcctccgagcggagtactgtcctccgagcggagtactgtcctccgagcggagtactgtcctccgagcggagtactgtcctccgagtggtctatataagcagagctcgttt agtgaaccgtcagatcgcctggagacgccatccacgctgttttgacctccatagaagac	S6
8x UAS for Gal4DBD binding + mini CMV promoter	cggagtactgtcctccgagcggagtactgtcctccgagcggagtactgtcctccgagcggagtactgtcctccgagcggagtactgtcctccgagcggagtactgtcctccgagcggagtactgtcctccgagcgga gtactgtcctccgagtggtctatataagcagagctcgtttagtgaaccgtcagatcgcctggagacgccatccacgctgttttgacctccatagaagac	S6

RT Primers	DNA sequence
RT-mKate-F	GGTGAACTTCCCATCCAACG
RT-mKate-R	ATGTCGCTTCTGCCTTCCAG
RT-GAPDH-F	GCACCACCAACTGCTTAGCC
RT-GAPDH-R	GGGCCATCCACAGTCTTCTG

Sequencing Primers	DNA sequence
SeqP-L4	CTTTGTATAGAAAAGTTG
SeqP-R1	TTTTTTGTACAAACTTGG
SeqP-L1	TACAAAAAAGCAGGCTGA
SeqP-L2	GTACAAGAAAGCTGGGTA
SeqP-R4	ACACAACATATCCAGTCACTATGG
SeqP-R2	CACTATGGTAATGCCAACTTTGTA
SeqP-Gb-g4-F	CCCTTATTAACGCGAGGGTAGTTG
SeqP-Gb-g7-F	GCCCTTATTAAAGTACGGGTCGTAC
SeqP-Gb-g10-F	GCCCTTATTAATACGAGGGCGATC
SeqP-Gb-gN-R	CTTGGGATCCGTCTTCTATGGAGG
SeqP-dCas1686F	TCAGACAGCAACTGCCTGAG
SeqP-dCas1889R	TGTCGAAAGTGCGCTGTTTG
SeqP-dCas5540F	GGCACACTGTCTGAAGCTCT
SeqP-dCas5994R	ATTCGTTTTGGCTGGCACAC
SeqP-dCas6476F	GCCACCAACTTCTCTCTGCT
SeqP-dCas6724R	TGCCTCGTCTTGCAGTTCAT

Linearization Primers	DNA sequence
pDEST1/2-R2-F	CCATAGTGACTGGATATGTTGTG
pDEST1/2-R4-R	CCATAGTGACTGGATATGTTG
pDEST1/2-g10-F	TACGAGGGCGATCCAACGAGTTTTAGAG
pDEST1/2-g10-R	TCCTGCCCGACCTTGGTACCG
pENTR-L4R1-R1-F	GGATCCCAAGGGCGAATTCGAC
pENTR-L4R1-L4-R	TTAATAAGGGCGAATTCGGAGCCAAC
pENTR-L1L2-L2-F	TACCCAGCTTTCTTGTACAAAGTTGG

pENTR-L1L2-L1-R	TCAGCCTGCTTTTTTGTACAAAGTTGG

Insert Cloning Primers	DNA sequence
g4/7/10-pDEST1/2-R4-F (overhang)	ATCCAGTCACTATGGTGTACAAAAAAGCAGGCTTTAAAGG
g4/7/10-pDEST1/2-R2-R (overhang)	ATCCAGTCACTATGGTAATGCCAACTTTGTACAAGAAAGCTGGG
g4-pENTR-L4-F (overhang)	GAATTCGCCCTTATTAACGCGAGGGTAG
g4-pENTR-R1-R (overhang)	TCGCCCTTGGGATCCGTCTTCTAT
g7-pENTR-L4-F (overhang)	GAATTCGCCCTTATTAAAGTACGGGTCGTA
g7-pENTR-R1-R (overhang)	TCGCCCTTGGGATCCGTCTTCTAT
g10-pENTR-L4-F (overhang)	GAATTCGCCCTTATTAATACGAGGGCGATC
g10-pENTR-R1-R (overhang)	TCGCCCTTGGGATCCGTCTTCTAT
CMV-pENTR-L4-F (overhang)	TTGACAACGCGGTGGTACTGAGTCATTAGGGACTTTCCAATGGG
CMV-pENTR-L4-R (overhang)	TCGCCCTTGGGATCCCCCAACAGTCGAGAGGTTTTCCG
AntiPD1_LC-L1-F (overhang+Kozak)	AAAAAAGCAGGCTGAGCCACCATGGGAGTTAAGGTATTGTTCGCATTGATTTGC
AntiPD1_LC-L2-R (overhang)	CAAGAAAGCTGGGTATTAGCATTCCCCTCGATTAAAGGACTTTG
AntiPD1_HC-L1-F (overhang+Kozak)	AAAAAAGCAGGCTGAGCCACCATGGGAGTTAAGGTATTGTTCGCATTGATTTGC
AntiPD1_HC-L2-R (overhang)	CAAGAAAGCTGGGTATTATTTACCGAGTGAAAGAGAGAGAGATTTCTG
dCas9-VPR-pENTR-L1-F (overhang)	TGTACAAAAAAGCAGGCTGAATGGACAAGAAGTACTCCATTGGGCT
dCas9-VPR-pENTR-L2-R (overhang)	TTGTACAAGAAAGCTGGGTATCAAAACAGAGATGTGTCGAAGATGGACAG
MS2/P65/HSF1-F (overhang)	TCCCAAGGGCGTACGGCCACCATGGCTTCAAACTTTACTCAG
MS2/P65/HSF1-R (overhang)	ATCCAGTCACTATGGTCTAGATCATTACTGCAGATCCTTTGCCCTAGG

Supplementary Table 2. List of plasmids used in experiments.

Name	Description	Purpose	First Appearance in Figure
pWC0001	pEF1a-mKate	positive control #1 constitutively expressing mKate	1
pWC0002	pCMV-mKate	positive control #2 constitutively expressing mKate	1
pWC0003	pEF1a-EBFP	constitutively expressing the transfection marker (EBFP)	1
pWC0011	pU6-sgRNA1	constitutively expressing gRNA1	1
pWC0012	pU6-sgRNA2	constitutively expressing gRNA2	1
pWC0013	pU6-sgRNA3	constitutively expressing gRNA3	1
pWC0014	pU6-sgRNA4	constitutively expressing gRNA4	1
pWC0015	pU6-sgRNA5	constitutively expressing gRNA5	1
pWC0016	pU6-sgRNA6	constitutively expressing gRNA6	1
pWC0017	pU6-sgRNA7	constitutively expressing gRNA7	1
pWC0018	pU6-sgRNA8	constitutively expressing gRNA8	1
pWC0019	pU6-sgRNA9	constitutively expressing gRNA9	1
pWC0020	pU6-sgRNA10	constitutively expressing gRNA10	1
pWC0021	p8xBS (sgRNA1)-mini-mKate	encoding the synthetic operator with 8 of gRNA1 BS to drive mKate expressior	1
pWC0022	p8xBS (sgRNA2)-mini-mKate	encoding the synthetic operator with 8 of gRNA2 BS to drive mKate expressior	1
pWC0023	p8xBS (sgRNA3)-mini-mKate	encoding the synthetic operator with 8 of gRNA3 BS to drive mKate expressior	1
pWC0024	p8xBS (sgRNA5)-mini-mKate	encoding the synthetic operator with 8 of gRNA5 BS to drive mKate expressior	1
pWC0025	p8xBS (sgRNA6)-mini-mKate	encoding the synthetic operator with 8 of gRNA6 BS to drive mKate expressior	1
pWC0026	p8xBS (sgRNA8)-mini-mKate	encoding the synthetic operator with 8 of gRNA8 BS to drive mKate expressior	1
pWC0027	p8xBS (sgRNA9)-mini-mKate	encoding the synthetic operator with 8 of gRNA9 BS to drive mKate expressior	S3
pWC0030	pEF1a-dCas9-VP16	constitutively expressing a dCas9-VP16	S3
pWC0031	pEF1a-dCas9-VP64	constitutively expressing a dCas9-VP64	S3
pWC0032	pEF1a-dCas9-VPR	constitutively expressing a dCas9-VPR	1
pWC0033	pCMV-dCas9-VPR	constitutively expressing a crisprTF	2
pWC0034	p2xBS (sgRNA4)-mini-mKate	encoding the synthetic operator with 2 of gRNA4 BS to drive mKate expressior	2
pWC0035	p4xBS (sgRNA4)-mini-mKate	encoding the synthetic operator with 4 of gRNA4 BS to drive mKate expressior	2
pWC0036	p6xBS (sgRNA4)-mini-mKate	encoding the synthetic operator with 6 of gRNA4 BS to drive mKate expressior	1
pWC0037	p8xBS (sgRNA4)-mini-mKate	encoding the synthetic operator with 8 of gRNA4 BS to drive mKate expressior	2
pWC0038	p12xBS (sgRNA4)-mini-mKate	encoding the synthetic operator with 12 of gRNA4 BS to drive mKate expressior	2
pWC0039	p16xBS (sgRNA4)-mini-mKate	encoding the synthetic operator with 16 of gRNA4 BS to drive mKate expressior	2
pWC0040	p2xBS (sgRNA7)-mini-mKate	encoding the synthetic operator with 2 of gRNA7 BS to drive mKate expressior	2
pWC0041	p4xBS (sgRNA7)-mini-mKate	encoding the synthetic operator with 4 of gRNA7 BS to drive mKate expressior	2
pWC0042	p6xBS (sgRNA7)-mini-mKate	encoding the synthetic operator with 6 of gRNA7 BS to drive mKate expressior	1
pWC0043	p8xBS (sgRNA7)-mini-mKate	encoding the synthetic operator with 8 of gRNA7 BS to drive mKate expressior	2
pWC0044	p12xBS (sgRNA7)-mini-mKate	encoding the synthetic operator with 12 of gRNA7 BS to drive mKate expressior	2
pWC0045	p16xBS (sgRNA7)-mini-mKate	encoding the synthetic operator with 16 of gRNA7 BS to drive mKate expressior	2
pWC0046	p2xBS (sgRNA10)-mini-mKate	encoding the synthetic operator with 2 of gRNA10 BS to drive mKate expressior	2
pWC0047	p4xBS (sgRNA10)-mini-mKate	encoding the synthetic operator with 4 of gRNA10 BS to drive mKate expressior	2
pWC0048	p6xBS (sgRNA10)-mini-mKate	encoding the synthetic operator with 6 of gRNA10 BS to drive mKate expressior	1
pWC0049	p8xBS (sgRNA10)-mini-mKate	encoding the synthetic operator with 8 of gRNA10 BS to drive mKate expressior	2
pWC0050	p12xBS (sgRNA10)-mini-mKate	encoding the synthetic operator with 12 of gRNA10 BS to drive mKate expressior	2
pWC0051	p16xBS (sgRNA10)-mini-mKate	encoding the synthetic operator with 16 of gRNA10 BS to drive mKate expressior	1
pWC0055	pEF1a-MS2-P65-HSF1	expressing synergistic activation mediator (SAM)	3
pWC0056	pU6-gRNA10-MS2H	expressing gRNA10 with MS2 hairpins	3
pWC0057	pU6-gRNA10-U6-gRNA10	dual gRNA transcriptional units	3
pWC0058	pCMV-2xNLS-dCas9-VPR	additional 2x nuclear localizing sequence (NLS) in the crisprTF	3
pWC0059	p4xBS (sgRNA4)-mini-SI-mKate	encoding the synthetic operator with 4 of gRNA4 BS to drive mKate expression with S	3
pWC0060	p8xBS (sgRNA4)-mini-SI-mKate	encoding the synthetic operator with 8 of gRNA4 BS to drive mKate expression with S	3
pWC0061	p12xBS (sgRNA4)-mini-SI-mKate	encoding the synthetic operator with 12 of gRNA4 BS to drive mKate expression with S	3
pWC0062	p16xBS (sgRNA4)-mini-SI-mKate	encoding the synthetic operator with 16 of gRNA4 BS to drive mKate expression with S	3
pWC0063	p4xBS (sgRNA7)-mini-SI-mKate	encoding the synthetic operator with 4 of gRNA7 BS to drive mKate expression with S	3
pWC0064	p8xBS (sgRNA7)-mini-SI-mKate	encoding the synthetic operator with 8 of gRNA7 BS to drive mKate expression with S	3
pWC0065	p12xBS (sgRNA7)-mini-SI-mKate	encoding the synthetic operator with 12 of gRNA7 BS to drive mKate expression with S	3
pWC0066	p16xBS (sgRNA7)-mini-SI-mKate	encoding the synthetic operator with 16 of gRNA7 BS to drive mKate expression with S	3
pWC0067	p4xBS (sgRNA10)-mini-SI-mKate	encoding the synthetic operator with 4 of gRNA10 BS to drive mKate expression with S	3
pWC0068	p8xBS (sgRNA10)-mini-SI-mKate	encoding the synthetic operator with 8 of gRNA10 BS to drive mKate expression with S	3
pWC0069	p12xBS (sgRNA10)-mini-SI-mKate	encoding the synthetic operator with 12 of gRNA10 BS to drive mKate expression with S	3
pWC0070	p16xBS (sgRNA10)-mini-SI-mKate	encoding the synthetic operator with 16 of gRNA10 BS to drive mKate expression with S	3
pWC0071	pattB-puro-U6-gRNA4-2xBS-mini-mKate-CMV-dCas9-VPR	payload for BxB1-mediated genomic integratior	4
pWC0072	pattB-puro-U6-gRNA4-4xBS-mini-mKate-CMV-dCas9-VPR	payload for BxB1-mediated genomic integratior	4
pWC0073	pattB-puro-U6-gRNA4-6xBS-mini-mKate-CMV-dCas9-VPR	payload for BxB1-mediated genomic integratior	4
pWC0074	pattB-puro-U6-gRNA4-8xBS-mini-mKate-CMV-dCas9-VPR	payload for BxB1-mediated genomic integratior	4
pWC0075	pattB-puro-U6-gRNA4-12xBS-mini-mKate-CMV-dCas9-VPR	payload for BxB1-mediated genomic integratior	4
pWC0076	pattB-puro-U6-gRNA4-16xBS-mini-mKate-CMV-dCas9-VPR	payload for BxB1-mediated genomic integratior	4
pWC0077	pattB-puro-U6-gRNA4-8xBS-mini-SI-mKate-CMV-dCas9-VPR	payload for BxB1-mediated genomic integratior	4

pWC0078	pattB-puro-U6-gRNA4-16xBS-mini-SI-mKate-CMV-dCas9-VPR	payload for BxB1-mediated genomic integratior	4
pWC0079	pattB-puro-U6-gRNA10-2xBS-mini-mKate-CMV-dCas9-VPR	payload for BxB1-mediated genomic integratior	4
pWC0080	pattB-puro-U6-gRNA10-4xBS-mini-mKate-CMV-dCas9-VPR	payload for BxB1-mediated genomic integratior	4
pWC0081	pattB-puro-U6-gRNA10-6xBS-mini-mKate-CMV-dCas9-VPR	payload for BxB1-mediated genomic integratior	4
pWC0082	pattB-puro-U6-gRNA10-8xBS-mini-mKate-CMV-dCas9-VPR	payload for BxB1-mediated genomic integratior	4
pWC0083	pattB-puro-U6-gRNA10-12xBS-mini-mKate-CMV-dCas9-VPR	payload for BxB1-mediated genomic integratior	4
pWC0084	pattB-puro-U6-gRNA10-16xBS-mini-mKate-CMV-dCas9-VPR	payload for BxB1-mediated genomic integratior	4
pWC0085	pattB-puro-U6-gRNA10-8xBS-mini-SI-mKate-CMV-dCas9-VPR	payload for BxB1-mediated genomic integratior	4
pWC0086	pattB-puro-U6-gRNA10-16xBS-mini-SI-mKate-CMV-dCas9-VPR	payload for BxB1-mediated genomic integratior	4
pWC0087	pattB-puro-U6-gRNA4-8xBS-mini-mKate-CMV-dCas9-VPR-2A-bla	payload for BxB1-mediated genomic integratior	4
pWC0088	pattB-puro-U6-gRNA4-16xBS-mini-mKate-CMV-dCas9-VPR-2A-bla	payload for BxB1-mediated genomic integratior	4
pWC0089	pattB-puro-U6-gRNA4-8xBS-mini-SI-mKate-CMV-dCas9-VPR-2A-bla	payload for BxB1-mediated genomic integratior	4
pWC0090	pattB-puro-U6-gRNA4-16xBS-mini-SI-mKate-CMV-dCas9-VPR-2A-bla	payload for BxB1-mediated genomic integratior	4
pWC0091	pattB-puro-U6-gRNA10-8xBS-mini-mKate-CMV-dCas9-VPR-2A-bla	payload for BxB1-mediated genomic integratior	4
pWC0092	pattB-puro-U6-gRNA10-16xBS-mini-mKate-CMV-dCas9-VPR-2A-bla	payload for BxB1-mediated genomic integratior	4
pWC0093	pattB-puro-U6-gRNA10-8xBS-mini-SI-mKate-CMV-dCas9-VPR-2A-bla	payload for BxB1-mediated genomic integratior	4
pWC0094	pattB-puro-U6-gRNA10-16xBS-mini-SI-mKate-CMV-dCas9-VPR-2A-bla	payload for BxB1-mediated genomic integratior	4
pWC0095	pattB-puro-dummyTU-EF1a-mKate-dummyTU	control payload for BxB1-mediated genomic integratior	4
pWC0096	pattB-puro-dummyTU-CMV-mKate-dummyTU	control payload for BxB1-mediated genomic integratior	4
pWC0100	pattB-hygro-CMV-dCas9-VPR-2A-G418	dLP1-1 payload constitutively expressing dCas9-VPR	5
pWC0200	pattB-puro-U6-gRNA10-8xBS-mini-mKate-8xBS-mini-TagBFP-EF1a-dCas9-VPR-2A-bla	dLP1-2 payload control expressing two reporters	5
pWC0201	pattB-puro-U6-gRNA10-16xBS-mini-SI-mKate-16xBS-mini-SI-TagBFP-EF1a-dCas9-VPR-2A-bla	dLP1-2 payload control expressing two reporters	5
pWC0202	pattB-puro-U6-gRNA10-8xBS-mini-JUG-LC-8xBS-mini-JUG-HC-EF1a-dCas9-VPR-2A-bla	dLP1-2 payload for JUG444 production (JUGAb1)	5
pWC0203	pattB-puro-U6-gRNA10-8xBS-mini-SI-JUG-LC-8xBS-mini-SI-JUG-HC-EF1a-dCas9-VPR-2A-bla	dLP1-2 payload for JUG444 production (JUGAb2)	5
pWC0204	pattB-puro-U6-gRNA10-16xBS-mini-JUG-LC-16xBS-mini-JUG-HC-EF1a-dCas9-VPR-2A-bla	dLP1-2 payload for JUG444 production (JUGAb3)	5
pWC0205	pattB-puro-U6-gRNA10-16xBS-mini-SI-JUG-LC-16xBS-mini-SI-JUG-HC-EF1a-dCas9-VPR-2A-bla	dLP1-2 payload for JUG444 production (JUGAb4)	5
pWC0206	pattB-puro-U6-gRNA10-8xBS-mini-PD1-LC-8xBS-mini-PD1-HC-EF1a-dCas9-VPR-2A-bla	dLP1-2 payload for anti-hPD1 production (PD1Ab1)	6
pWC0207	pattB-puro-U6-gRNA10-8xBS-mini-SI-PD1-LC-8xBS-mini-SI-PD1-HC-EF1a-dCas9-VPR-2A-bla	dLP1-2 payload for anti-hPD1 production (PD1Ab2)	6
pWC0208	pattB-puro-U6-gRNA10-16xBS-mini-PD1-LC-16xBS-mini-PD1-HC-EF1a-dCas9-VPR-2A-bla	dLP1-2 payload for anti-hPD1 production (PD1Ab3)	6
pWC0209	pattB-puro-U6-gRNA10-16xBS-mini-SI-PD1-LC-16xBS-mini-SI-PD1-HC-EF1a-dCas9-VPR-2A-bla	dLP1-2 payload for anti-hPD1 production (PD1Ab4)	6
pWC0300	pattB-puro-U6-gRNA10-MS2H-8xBS-mini-mKate-CMV-dCas9-VPR-2A-bla-EF1a-MS2-P65-HSF1-2A-hygrc	payload for BxB1-mediated genomic integratior	S13
pWC0301	pattB-puro-U6-gRNA10-MS2H-16xBS-mini-SI-mKate-CMV-dCas9-VPR-2A-bla-EF1a-MS2-P65-HSF1-2A-hygrc	payload for BxB1-mediated genomic integratior	S13
pWC0900	pCMV-Gal4-VPR	constitutively expressing Gal4-VPR	S6
pWC0901	p2xUAS-BS-mini-mKate	encoding the synthetic operator with 2 of UAS BS to drive mKate expressior	S6
pWC0902	p4xUAS-BS-mini-mKate	encoding the synthetic operator with 4 of UAS BS to drive mKate expressior	S6
pWC0903	p6xUAS-BS-mini-mKate	encoding the synthetic operator with 6 of UAS BS to drive mKate expressior	S6
pWC0904	p8xUAS-BS-mini-mKate	encoding the synthetic operator with 8 of UAS BS to drive mKate expressior	S6
